# Water removal during automated peritoneal dialysis assessed by remote patient monitoring and modelling of peritoneal tissue hydration

**DOI:** 10.1038/s41598-021-95001-x

**Published:** 2021-08-02

**Authors:** Joanna Stachowska-Pietka, Beata Naumnik, Ewa Suchowierska, Rafael Gomez, Jacek Waniewski, Bengt Lindholm

**Affiliations:** 1 Nalecz Institute of Biocybernetics and Biomedical Engineering, Polish Academy of Sciences, 4 Trojdena Str, 02-109 Warsaw, Poland; 2 The First Department of Nephrology and Transplantation with Dialysis Unit, Medical University of Bialystok, Bialystok, Poland; 3 RTS, Cali, Colombia; 4 Division of Renal Medicine and Baxter Novum, Department of Clinical Science, Intervention and Technology, Karolinska Institutet, Stockholm, Sweden

**Keywords:** Renal replacement therapy, Applied mathematics

## Abstract

Water removal which is a key treatment goal of automated peritoneal dialysis (APD) can be assessed cycle-by-cycle using remote patient monitoring (RPM). We analysed ultrafiltration patterns during night APD following a dry day (APD_DD_; no daytime fluid exchange) or wet day (APD_WD_; daytime exchange). Ultrafiltration for each APD exchange were recorded for 16 days using RPM in 14 patients. The distributed model of fluid and solute transport was applied to simulate APD and to explore the impact of changes in peritoneal tissue hydration on ultrafiltration. We found lower ultrafiltration (mL, median [first quartile, third quartile]) during first and second vs. consecutive exchanges in APD_DD_ (−61 [−148, 27], 170 [78, 228] vs. 213 [126, 275] mL; p < 0.001), but not in APD_WD_ (81 [−8, 176], 81 [−4, 192] vs. 115 [4, 219] mL; NS). Simulations in a virtual patient showed that lower ultrafiltration (by 114 mL) was related to increased peritoneal tissue hydration caused by inflow of 187 mL of water during the first APD_DD_ exchange. The observed phenomenon of lower ultrafiltration during initial exchanges of dialysis fluid in patients undergoing APD_DD_ appears to be due to water inflow into the peritoneal tissue, re-establishing a state of increased hydration typical for peritoneal dialysis.

## Introduction

In peritoneal dialysis (PD), efficient removal of excess water by peritoneal ultrafiltration is a key treatment goal and a predictor of patient survival^1^. Peritoneal ultrafiltration depends on interactions between dialysis fluid and the peritoneal tissue in which embedded blood capillaries and lymphatics form a complex peritoneal transport system, usually denoted peritoneal membrane, serving as a natural dialysis filter. Various mathematical methods have been used to explore the complex relations between net ultrafiltration and peritoneal tissue properties^2–5^.

Net ultrafiltration—which is a function of transcapillary ultrafiltration, driven by the osmotic force induced by hypertonic dialysis fluid, and water reabsorption from the peritoneal cavity—is influenced by factors such as infused volume, the effective peritoneal surface area in contact with dialysis fluid and exposure time of dialysis fluid in full contact with the peritoneal membrane. These factors vary considerably in automated peritoneal dialysis (APD). APD is typically performed during the night, preceded either by a “dry day” (APD_DD_) without intraperitoneal dialysate for the whole daytime (except for the residual volume remaining after drainage), or by a “wet day” (APD_WD_) with dialysate left in the peritoneal cavity for a single or sometimes two long daytime dwells. Water removal during each APD exchange might be expected not to differ if prescriptions of volume infused, dwell time, and glucose concentrations remain constant, but it is not known if the 12- to 14-h rest of peritoneum in APD_DD_ could influence peritoneal tissue properties and thereby ultrafiltration during the following APD session.

Peritoneal tissue properties may change during PD because hydrostatic and osmotic pressures of the fluid infused into the peritoneal cavity are considerably higher than corresponding pressures in the peritoneal tissue´s interstitial fluid, leading to increased hydration of the tissue close to the peritoneal surface, as shown experimentally^6^ and by mathematical modelling of clinical data^2,7^. During APD_WD_, with fluid remaining in the peritoneal cavity during daytime, peritoneal tissue hydration could be assumed to remain increased whereas during the long daytime dwell in APD_DD_, the hydrostatic pressure and hydration of the peritoneal tissue may conceivably decrease towards physiological levels.

Net ultrafiltration and other aspects of APD treatment can be studied in detail using a new generation of cyclers equipped with device for remote patient monitoring (RPM), which collects dialysis treatment data that can be used to improve patient care by checking adherence to and modifying dialysis prescriptions^8–10^. The advent of APD with RPM allows for systematic monitoring of the kinetics of infused and drained dialysate volume, and therefore also of the net ultrafiltration (UF) for each exchange (cycle) of the APD session.

The aim of this study was to explore possible effects of the preceding day’s exchange on net ultrafiltration of the first exchanges of the subsequent APD session. We first investigated if water removal differed between initial and subsequent consecutive APD exchanges in patients on wet and dry day regimes using data collected by RPM. We then attempted to provide a physiological explanation of observed differences of peritoneal fluid transport during APD cycles and the daytime exchange using numerical simulations from mathematical modelling.

## Materials and methods

### Patients

In this observational retrospective study, data on ultrafiltration were collected and analysed in patients undergoing APD at the First Department of Nephrology and Transplantation, Medical University of Bialystok, Bialystok, Poland. After excluding patients with variable tonicity of dialysis fluid infused during consecutive night exchanges to avoid the impact of dialysate tonicity on ultrafiltration during initial APD cycles, we included data from 14 patients median age 31.5 (range, 24 to 77) years; seven women; preceding median time on dialysis was 13 (range, 4 to 60) months in the present study. Three patients were high transporters (H), nine high-average transporters (HA), and two low-average transporters (LA) according to peritoneal equilibration test (PET). In all patients, net ultrafiltration was investigated using RPM data for each exchange during 16 consecutive days. Six patients received APD with a dry day regime (APD_DD_) and 8 patients had a wet day regime (APD_WD_), see Table 1. Clinical characteristics were similar (p ≥ 0.5; Mann–Whitney U test) albeit with tendency for faster small solute transport rate in APD_DD_ vs. APD_WD_ (p > 0.1 for PET D/P_creat_). No clinical evidences of presence of systemic edema or changes of the fluid status were reported during the investigated time period.

**Table 1:** Clinical characteristics of 14 patients undergoing APD with remote patient monitoring allowing recording of volumes of infusion and drainage of dialysate; patients were undergoing APD with wet day regime (APD_WD_) or dry day regime (APD_DD_).

Clinical characteristics	APD_WD_ n = 8	APD_DD_ n = 6
Sex, women/men	4/4	3/3
Age, years	37 (24–77)	31.5 (24–66)
Body weight, kg	65.1 (51–110)	67 (38–112.9)
Diastolic blood pressure, mmHg	86 (67–90)	78 (70–110)
Systolic blood pressure, mmHg	138 (117–166)	138 (124–155)
PET D/P_creat_	0.74 (0.66–0.77)	0.80 (0.72–0.92)
Dialysis vintage, months	12 (4–60)	13 (10–55)
Diuresis, mL	600 (0–2100)	1150 (0–2000)

Data are median (min–max) if not stated otherwise. For blood pressure and body weight, the mean values for each patient over 16 days were noted.

*PET D/P*_*creat*_ - Peritoneal Equilibration Test dialysate-to-plasma concentration of creatinine.

The study was conducted according to the principles of Declaration of Helsinki. Ethical approval for this study was obtained from Bioethical Commission of the Medical University of Bialystok (APK.002.225.2020). Informed consent to use retrospective routine clinical data and to publish anonymized data was obtained from the patients.

All patients used APD with RPM (HomeChoice Claria with the Sharesource connectivity platform; Baxter Healthcare Corporation, Deerfield, Il, USA). For night APD exchanges, the patients used glucose-based dialysis fluid (Dianeal or Physioneal with glucose 1.36% or 2.27%, Baxter, Castlebar, Ireland), and for daytime exchanges, icodextrin-based (Extraneal, Baxter, Castlebar, Ireland) or glucose-based (Physioneal or Dianeal glucose 1.36% or 2.27%) solution; see Table 2. The number of night cycles varied between patients from 4 to 7 with median 5 cycles in both groups. There were no statistically significant differences between APD_WD_ and APD_DD_ group with respect to infused volume, number of cycles per APD session, or total glucose load for night APD session; however, as expected, infused volume for daytime exchanges and the glucose/carbohydrate load were higher (p = 0.002) in APD_WD_ group. For each patient, the net ultrafiltered volume, measured as a difference between infused and drained volume, were noted for each exchange by the cycler, each day for 16 consecutive days.

**Table 2:** Characteristics of automated peritoneal dialysis (APD) schemes prescribed for 8 patients on wet day (APD_WD_) and for 6 patients with dry day (APD_DD_) regimes and comparison of APD_WD_ with APD_DD_ group.

Dialysis exchanges	APD_WD_ n = 8	APD_DD_ n = 6
**Night APD exchanges**		
*Fluid type*		
Glucose 1.36%	50%	50%
Physioneal 1.36%	25%	33%
Dianeal 1.36%	25%	17%
Glucose 2.27%	50%	50%
Physioneal 2.27%	44%	50%
Dianeal 2.27%	6%	0%
Total number of cycles	5 (4–6)	5 (4–7)
Volume infused per cycle, mL	1850 (1600–2000)	1800 (1600–2300)
Glucose load, g/session	152 (109–259)	165 (116–254)
**Day exchange**		
*Fluid type*		
Glucose 1.36%	13%	50%
Physioneal 1.36%	13%	33%
Dianeal 1.36%	0%	17%
Glucose 2.27%	0%	50%
Physioneal 2.27%	0%	50%
Dianeal 2.27%	0%	0%
Icodextrin (extraneal) 7.5%	87%	0%
Volume infused, mL	1450 (1000–1800)	100 (100–500)**
Carbohydrate/glucose load, g/exchange	109 (14–135)	2 (1–7)**

Data are presented as percentage, or median (min–max).

**p = 0.002; difference APD_WD_ vs. APD_DD_ (Mann–Whitney U test). In APD_DD_ group, 4 patients received 100 mL, 1 patient 300 mL and 1 patient 500 mL of glucose 1.36% dialysis fluid for day exchange (to avoid pain during the daytime and subsequent first APD exchange).

As a routine procedure, to avoid pain associated with low fluid volume in the peritoneal cavity after day dwell in patients on the APD_DD_ regime, a small volume of dialysis fluid (minimum of 100 mL, up to 500 mL in some APD_DD_ patients) was infused after the last night APD cycle.

### Calculations

Data on infused and drained volume from each APD cycle and day exchange were analysed for each patient over a period of 16 days. The values of the net ultrafiltration obtained after first (C1) and second (C2) cycle were compared with the mean ultrafiltration volume from remaining consecutive cycles (cycle 3 and subsequent cycles, maximally up to 7), denoted by C3^+^. For each patient, net UF was calculated for night and day sessions separately and for the whole 24-h period. Daily water removal was calculated as the sum of net UF from night and day exchanges, plus daily diuresis. In one patient, one measurement of ultrafiltration from a day exchange was excluded due to an abnormal value.

Because dialysis fluid tonicity differed between patients from the two study groups, and drained volume depends on the glucose concentration in dialysate, we performed additional analyses of drain volume from first (VdrR_1_) or second (VdrR_2_) exchange over mean drain volume from all remaining exchanges, UF_C3+,_ calculated as: VdrR_*Ci*_ = UF_Ci_/UF_C3+_, where *i* stands for first or second cycle.

### Peritoneal transport model

The spatially distributed model that considers tissue hydration status^7^ was applied to simulate peritoneal transport during APD_DD_; see Supplementary data for details**.** In brief, computer simulations were performed for a typical patient undergoing standard APD with six 90 min cycles with infused volume of 2 L of glucose 1.36% followed by dry day regime with infused volume of 100 mL of glucose 1.36%.

### Statistical methods

The differences among ultrafiltration volumes after first, second and remaining cycles were analysed using repeated measures analysis of variance (ANOVA) with multivariate tests. If ANOVA showed a significant difference, the post-hoc Tukey’s test was applied. The differences between the two variables were checked using Student t-test, Mann–Whitney U test, or Wilcoxson tests, as appropriate. The statistical significance level was set at p = 0.05, and data were presented as median [first quartile Q1, third quartile Q3], if not stated otherwise.

## Results

The characteristics of water removal were calculated for each patient undergoing APD_DD_ and APD_WD_ for each exchange, for 16 days. Due to high day-to-day variability of UF volumes for essentially all patients, we report median UF volumes calculated for each patient for the whole period of 16 days (Table 3). No significant differences were found between APD_DD_ and APD_WD_ concerning the night and day UF, total peritoneal net UF, diuresis and total 24 h water removal (p > 0.1; Mann–Whitney U test).

**Table 3:** The overall water removal (net UF, median [Q_1_, Q_3_] in mL) from night APD exchanges and the long daytime exchange, diuresis and total 24 h water removal, measured in patients undergoing APD with wet day (APD_WD_) or a dry day regime (APD_DD_).

Water removal, mL	APD_WD_ n = 8	APD_DD_ n = 6
Night net UF	425 [132, 838]	565 [453, 889]
Day net UF	254 [−80, 471]	−96 [−166, −91]
Total peritoneal net UF	813 [123, 1067]	472 [356, 723]
Diuresis	600 [0, 1550]	1150 [775, 1825]
Total 24 h water removal	1592 [1314, 1806]	1771 [1431, 2062]

No significant differences were found between APD_DD_ and APD_WD_ concerning the night and day net UF, total peritoneal net UF, diuresis and total 24 h water removal (p > 0.1; Mann–Whitney U test).

**Figure 1:**
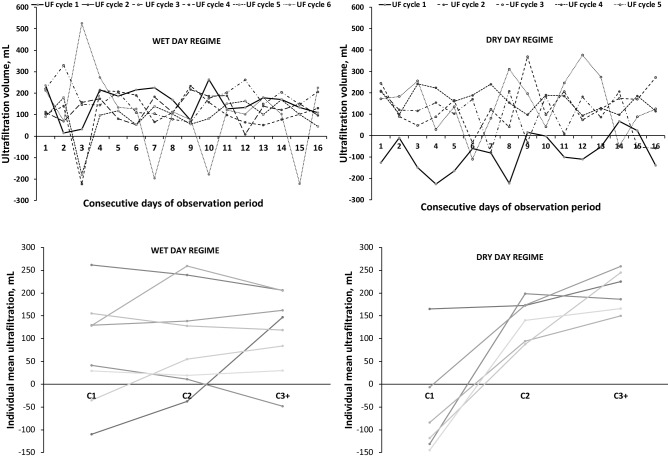
Ultrafiltration patterns during APD according to data delivered by continuous remote monitoring. Upper panel: Net ultrafiltration volume (in mL) of each APD exchange during the observation period of 16 days for two patients undergoing APD with wet day (APD_WD_ with 6 night exchanges) and dry day (APD_DD_ with 5 night exchanges) regimes respectively. Net UF from each cycle/exchange of the APD session is denoted by a different colour. The bold, solid line corresponds to net UF from first cycle obtained during consecutive days. Bottom panel: Individual values of mean net ultrafiltration (in mL) in the first (C1), and second (C2) cycle, and the mean values of the following cycles (C3 +) during the observation period of 16 days for each of the 14 patients undergoing either APD_WD_ (n = 8) or APD_DD_ (n = 6).

In patients on APD_DD_, but not in those receiving APD_WD_, the initial cycle was consistently associated with lower net UF compared with subsequent cycles (Fig. 1, upper panel). To better illustrate this phenomenon, the mean values (over days) of net UF observed in the first (C1), second (C2), and the following cycles (C3 +) for each patient from APD_DD_ and APD_WD_ group are shown in Fig. 1, bottom panel.

A statistically significant dependence on cycle number was found in the APD_DD_ group (p < 0.001; ANOVA repeated measurements test) whereas inter-cycle differences of net UF were not statistically significant in APD_WD_. Further post-hoc analysis showed significantly lower UF in the APD_DD_ group for C1 vs. C2 and for C1 vs. C3 + (p ≤ 0.001), Table 4.

**Table 4:** Net peritoneal ultrafiltration (UF, median [Q_1_, Q_3_]) measured for cycle 1 (C1), cycle 2 (C2) and for the mean value from cycle 3 and following cycles (C3 +), as well as the ratio of drain volumes calculated for first (VdrR1) and second (VdrR2) cycle over a mean value for C3 + for patients undergoing APD_WD_ and APD_DD_.

Net UF and drain volume ratio	APD_WD_ n = 8	APD_DD_ n = 6
UF in C1, mL	81 [−8, 176]	−61 [−148, 27]
UF in C2, mL	81 [−4, 192]	170 [78, 228]*
Mean UF in C3 + , mL	115 [4, 219]	213 [126, 275]**
VdrR1	1.00 [0.93, 1.04]	0.87 [0.81, 0.93]^##,†^
VdrR2	1.00 [0.94, 1.04]	0.97 [0.92, 1.00]^#, †^

*p ≤ 0.001; Tukey’s post-hoc test comparing C1 vs. C2,

**p ≤ 0.001; Tukey’s post-hoc test comparing C1 vs. C3 + ,

^#^p < 0.05; Wilcoxon comparing median values of VdrR1 with VdrR2,

^##^p < 0.05; t-test comparing median values of VdrR for APD_WD_ vs. APD_DD_,

^†^p < 0.05; Wilcoxon to test if the value is lower than 1.

Median values of drain volume ratios were below unity in APD_DD_ (VdrR1 = 0.87 and VdrR2 = 0.97) but not in APD_WD_ patients (VdrR1 = 1.00 and VdrR2 = 1.00). The individual values of VdrR1 and VdrR2 (for all days of the observation period) were significantly lower than one in APD_DD_ (both p < 0.001) but not in APD_WD_ patients (p = 0.11 and p = 0.25, respectively). Moreover, in APD_WD_, median values of VdrR1 vs. VdrR2 did not differ (p = 0.73) whereas this difference was significant for APD_DD_ (p = 0.03). Finally, VdrR differed between APD_WD_ and APD_DD_ for VdrR1 (p < 0.001), but not for VdrR2 (p = 0.10), Table 4.

**Figure 2:**
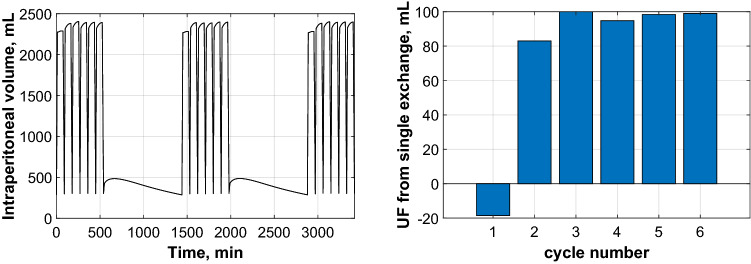
Numerical simulations for a typical APD_DD_ patient showing negative net UF during the first APD cycle in contrast to positive net UF during the consecutive five cycles. Intraperitoneal volume profiles as a function of time during three consecutive APD sessions (consisting of six 90-min exchanges with 2 L of glucose 1.36%) interrupted by dry days (with infusion of 100 mL of glucose 1.36%) (left panel) and the corresponding net UF per each of the 6 single exchanges of the last (third) day’s APD session (right panel).

To explore if lower net UF during initial cycles in the APD_DD_ group could be attributed to altered tissue hydration status, data were analysed using the spatially distributed model^2,7^. Numerical simulations for a typical APD_DD_ patient showed negative net UF during the first APD cycle in contrast to positive net UF during the consecutive five cycles, Fig. 2.

The distributed model predicted total net UF for the whole APD session of 458 mL, negative ultrafiltration of -19 mL during first APD cycle, 83 mL in C2, and mean ultrafiltration of 98 ± 3 mL during remaining cycles, see Table 5. Net UF for the first APD exchange (C1:UF = —19 mL) was 114 mL lower than the mean value of net UF from subsequent five cycles, Fig. 2 and Table 5.

**Table 5:** Volumes of water removal predicted by the distributed model: net UF for the whole APD session, first (C1) and second (C2) APD cycle and mean ± SD net UF in consecutive cycles (C3^+^), and water accumulation in the peritoneal tissue during the first cycle (C1) and during first two cycles (C1 plus C2) for a typical patient undergoing APD with a dry day regime (APD_DD_) with infusion of 100 mL of glucose 1.36% during the day to prevent pain.

Fluid removal and fluid accumulation in the tissue	APD_DD_ with Vinf = 100 mL
UF for APD session, mL	458
UF in C1, mL	−19
UF in C2, mL	83
Mean UF in C3 + , mL	98 ± 3
Water accumulated in C1, mL	187
Water accumulated in C1 and C2, mL	269

**Figure 3:**
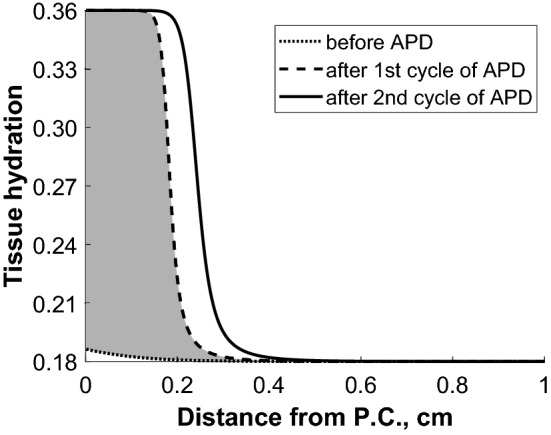
Predictions by the applied distributed model^2, 7^ of peritoneal tissue hydration as a function of distance from the peritoneal cavity (P.C.) and changes during APD with dry day followed by night APD exchanges (APD_DD_ regime). Dotted line—before starting of APD session; dashed line—after the first cycle of APD session; solid line—after the second cycle of APD session. Before starting of APD session, the tissue hydration profile is slightly above the physiological level of tissue hydration of 0.18 (18%) that is typically kept in deeper tissue layers. The shadowed area denotes the change in tissue hydration occurring during the first cycle of APD session due to water absorbed into the peritoneal tissue driven by the high intraperitoneal pressure gradient following the infusion of dialysis fluid. The APD_DD_ session was simulated with 6 cycles of 90 min each with an infusion of 2 L of glucose 1.36% preceded by a “dry” daytime exchange with an infusion of 100 mL of glucose 1.36%.

According to the applied model^2,7^, during PD, the inflow of fluid and solutes into the peritoneal tissue, induced by the osmotic force, results in a local increase in interstitial hydrostatic pressure that increases tissue hydration above the physiological level of about 18% in the layers close to the peritoneal cavity but gradually decreases as a function of increasing distance from the peritoneal cavity, see Fig. 3. In patients on APD_WD_, peritoneal tissue hydration in the layers close to the peritoneal cavity remains higher than the physiological state of hydration throughout 24 h because of the unphysiological presence of dialysis fluid. In contrast, in patients on the APD_DD_ regime, peritoneal tissue hydration is not constant. After a dry day with almost empty peritoneal cavity, peritoneal tissue hydration at the start of the APD session is only slightly above the physiological hydration of about 18%, see Fig. 3. The inflow of dialysis fluid at the start of the APD session results in an immediate increase of tissue hydration close to the peritoneal cavity with a further increase after the second exchange resulting in a state of tissue overhydration that is typical in PD, Fig. 3. The increase in tissue hydration during the first APD cycle was caused by inflow of 187 mL of water accumulating in the peritoneal tissue, and by a total accumulation of 269 mL during the two initial exchanges of APD session, respectively.

## Discussion

Our analysis of UF data delivered by RPM in APD patients treated at home revealed lower net UF during the first cycle of APD session in patients using a dry day regime (APD_DD_) as compared to those on a wet day regime (APD_WD_); and some APD_DD_ patients had negative net UF during the first APD exchange. Peritoneal transport modelling indicated that a likely explanation for this phenomenon is that the daytime exchange influenced the hydration state of the peritoneal membrane and that this affected water removal during the initial exchange(s) of the subsequent APD session. Thus, the observed initial lower efficiency of water removal in patients on the APD_DD_ regime appears to be related to a relative increase of peritoneal tissue hydration during the first APD cycles following a dry day.

The acquisition of clinical data relied on the advent of RPM, which was designed to track, on a daily basis, factors such as blood pressure, body weight, dialysis treatment characteristics including ultrafiltration, and patient´s adherence to dialysis prescriptions in patients undergoing APD at home^8^, and which offers unique opportunities for studies of net UF of APD exchanges cycle-by-cycle. To the best of our knowledge, this is the first detailed study of UF patterns during APD using this technology.

Lower UF volumes during the initial as compared to subsequent cycles was demonstrated in APD_DD_ but not in APD_WD_ when analysing drain volume ratios (of initial cycle volume to subsequent cycle volumes): in APD_DD_, but not in APD_WD_, VdrR1 was lower than VdrR2 and both ratios were significantly lower than 1 (Table 4). Moreover, VdrR was lower in APD_DD_ than in APD_WD_ indicating that the daytime exchange influenced water removal during the subsequent APD_DD_ session. We have not identified any published studies describing this phenomenon.

To further explore why APD_DD_ patients had lower net UF during the first cycles of APD, we performed numerical simulations based on the distributed model^2,7^ for a typical APD_DD_ patient with standard prescription (vide supra, and Table 2). These simulations provided results which fit well with clinical data. Thus, while the simulated net UF of the daytime exchange was -110 mL and 458 mL for the whole night APD session, the corresponding values in the subgroup of APD_DD_ patients (with glucose 1.36% prescribed) was -188 mL and 440 mL respectively.

Using the applied model, we explored changes in the hydration status of the peritoneal tissue, which is typically not considered in physiological interpretations, and in most mathematical models of the peritoneal transport it is assumed constant^3,7,11,12^. However, water reabsorption from the peritoneal cavity (driven by the high intraperitoneal pressure gradient) to the adjacent tissue layers may correspond to more than 70% of total peritoneal fluid absorption^13^. The water inflow into the peritoneal tissue induces an increase of interstitial pressure and tissue hydration over their physiological levels. Such an increase, observed also in inflammation, facilitates water and solute transport by changing transport properties of the peritoneal membrane. During the treatment, the interstitial pressure close to the peritoneal cavity increases to equilibrate with the intraperitoneal pressure. Such an increase of interstitial pressure and the corresponding increase in tissue hydration in the layer close to the peritoneal cavity is predicted by the applied distributed model but was also observed experimentally in an animal model of PD and in basic physiology studies^2,6,7,11,14,15^.

Numerical simulations were performed for the standard distributed model that takes into account the spatial character of the peritoneal barrier and was validated previously based on clinical and experimental data. In the model, the *functional relation* between dynamic changes of the tissue hydration and interstitial pressure and their impact on the local transport properties were considered (based on the experimental data). Analysis based on this model showed increase of tissue hydraulic conductivity and solute diffusivity close to the peritoneal cavity (within 0.2 cm distance from the peritoneal surface) caused by the local increase of tissue hydration and interstitial pressure or local increase of tissue lymphatic absorption or permeability caused by the local interstitial pressure increase (as a part of edema preventing mechanism). All these mechanisms have been investigated experimentally previously and corresponding functional relations were validated in the earlier versions of the model and were used in the present version of the model, see^2,6,7,11,16–19^. It should be noted that the increase of tissue hydration, observed during the peritoneal dwell, is restricted to the thin layer of 0.2 cm width measured from the peritoneal surface, see Fig. 3, and does not reflect changes in the deeper tissue layers or in the fluid status of the whole body where hydration may remain unchanged. Moreover, the spatial character of the barrier results in a non-constant glucose profile across the peritoneal barrier (exponentially decaying, see for example Flessner et al.^20^) that has local vasodilatory effect on tissue blood capillaries situated within short distance from the peritoneal cavity. The detailed mechanism of the vasodilation in case of PD has been investigated experimentally by Zakaria et al.^21–25^ and the functional relation was implemented in the currently applied model that considers changes in the effective surface area of the blood capillary wall without detailed modelling of mechanisms. One may also speculate that high glucose concentration, as present in the dialysis fluid and locally in the tissue close to the peritoneal cavity, may lead to the local increase of AQP-1 expression as has been shown in *in vitro* studies for endothelial and mesothelial cells by Lai et al.^26^ and modelled using the three-pore model by Stachowska-Pietka et al.^27^. This would result in similar changes as those induced by local vasodilation. Nevertheless, numerical simulation of the DD cycles showed that although the same functional relations of physiological properties were assumed, the differences in tissue hydration between cycles led to the different local tissue properties and, in consequence, different treatment effectiveness. During a dry day regime (APD_DD_), when the peritoneal cavity is empty or almost empty, intraperitoneal pressure remains lower than the interstitial pressure in the adjacent tissue layers, leading to slow leakage of water accumulated in the tissue into the peritoneal cavity. Consequently, peritoneal tissue hydration decreases to the physiological (lower) state of tissue hydration. At the beginning of APD_DD_ session, the infusion of hypertonic dialysis fluid not only induces ultrafiltration into the peritoneal cavity due to the osmotic force. Besides, a part of the water remains in the tissue and increases its hydration, adapting to the PD condition by equilibrating with the fluid in the peritoneal cavity. In contrast, during a wet day regime (APD_WD_), tissue hydration during the day remains unphysiologically high (as is typical for continuous forms of PD) due to the presence of dialysate throughout the day, resulting in elevated intraperitoneal pressure during daytime. Therefore, any decrease of tissue hydration after a daytime exchange is negligible and there is only a minor (within measurement error) impact on water removal during the subsequent initial APD cycles.

Some strengths and limitations of the study should be noted. Strengths include the availability of detailed data of net UF cycle-by-cycle provided by RPM in two similar groups of patients using the two APD regimes and the long observation period (16 days) in each patient. However, one cannot exclude that at least part of the UF variability between cycles, as observed in our study, might be related also to other factors that are not considered in the applied model, such as changes in the residual peritoneal volume (due to differences in drainage), physical activity of the patients that influences intraperitoneal pressure (such as coughing, patient’s posture during drainage), diuresis, and overall volume status. Therefore, in contrast to solute removal which can be predicted very precisely by available peritoneal transport models, peritoneal ultrafiltration is difficult to predict.

In general, the residual volume may differ not only between patients but also between APD cycles, having an impact on the evaluation on the water removal from each exchange. The impact of possible differences in residual volumes before each APD cycle might be especially important in case of APD with low glucose solution, due to the relatively small ultrafiltered volume per cycle. The precise evaluation of the residual volume would require additional procedures performed before each cycle such as rinse procedure (to evaluate residual volume from dilution technique) or usage of a volume marker, that were outside the scope of the study. Therefore, for the purpose of this study, it was assumed that the residual volume is not changing between cycles of APD sessions for a particular patient. Moreover, we assumed that the impact of the difference in intraperitoneal volume remaining after daytime exchange on the initial drainage before APD session (DD vs WD regime) is compensated by the default settings of the cycler (setting the same initial draining force, and automatically stopping drainage if the fluid flow rate is too low) as suggested by our data. We found that the average amount of drained volume in the group of APD_WD_, 1812 [2111–1270] ml (median value with range [Q3–Q1]), was significantly higher than the corresponding value for APD_DD_ group, 27 [101–4] mL. Nevertheless, this would not influence our findings since they were mostly based on the analysis comparing cycles within the same patient and for the same APD regime. The only comparison between the two regimes was done based on the drained volumes ratio, VdrR (Table 4), which showed significant difference between the two APD regimes even when excluding the first cycle (VdrR2) and after correcting for fluid tonicity.

The low number of patients in each of the studied groups is another limitation; further investigations of larger cohorts of patients are warranted to confirm our findings. One problem with the selection of patients for this kind of clinical study is that many patients using cyclers change glucose concentration of dialysis fluid cycle-to-cycle during the night exchanges due to changed prescription, and therefore only a rather low number of patients use the same glucose concentration in all cycles. In our study only patients using dialysis fluid with constant glucose concentration were investigated. Moreover, although both studied groups had statistically similar characteristics, there was a tendency for faster transport status in APD_DD_ than in APD_WD_ group, that might have a slight effect on the comparison of both groups but not the results in each group separately. Furthermore, a difference between the groups is that all but one of the APD_WD_ patients used icodextrin-based dialysis fluid during the long day dwell that significantly increases net UF^28–32^ and may have a different impact on peritoneal tissue hydration. Nevertheless, we believe that presented results supported by the mathematical modelling for a typical patient indicate a possible reason for at least part of the observed variability in the water removal between consecutive APD cycles. It should be noted however that there was no difference between the two APD regimes concerning the total obtained volume of net UF from the night and day exchanges and 24 h UF, Table 3. Moreover, although we observed lower efficiency of water removal during initial APD_DD_ cycles in our patients, this effect was relatively minor from a clinical point of view. However, the role of this phenomenon in other situations—such as in case of adapted APD schedules with variable dwell time being combined with variable dwell volumes^33^, use of different glucose concentrations, or in case of patients with different transport characteristics or different diuresis—is not clear and needs further investigations.

In summary, our investigation of ultrafiltration cycle-by-cycle during APD assessed by remote monitoring of patients treated at home revealed that compared with a “wet day” APD regime (APD_WD_), patients on a “dry day” APD regime (APD_DD_) had lower water removal during the first as compared to subsequent APD cycles, and in some cases even negative UF. According to peritoneal transport modelling, the observed difference in initial UF between the two regimes may reflect local adaptation of the peritoneal tissue to the presence (APD_WD_) vs. absence (APD_DD_) of dialysis fluid in the peritoneal cavity during the long daytime dwell. During the dry day (APD_DD_), peritoneal tissue hydration decreases towards a physiological level of about 18% and then increases during the first APD cycle to about 36% (in tissue layer close to the peritoneal cavity), a level typical for conditions with continuous presence of dialysis fluid such as in APD_WD_. We conclude that the increase of peritoneal tissue hydration during the first APD cycle in APD_DD_ patients appears to be a consequence of inflow of water into the peritoneal tissue—thereby reducing the inflow into the peritoneal cavity—resulting in a corresponding decrease of net UF.

## Supplementary Information

Supplementary Information.

## Data Availability

A detailed description of the mathematical model used in this study and values of all model parameters may be found in the Supplementary material and in our previous work referenced in the manuscript. Anonymized clinical data are available on request.
