# Supplementary material for: Water removal during automated peritoneal dialysis assessed by remote patient monitoring and modelling of peritoneal tissue hydration
Source: Sci Rep. 2021 Aug 2;11:15589. doi: 10.1038/s41598-021-95001-x (PMC8329227; doi:10.1038/s41598-021-95001-x)
Supplement: Supplementary file 1 — Supplementary Information. [file 41598_2021_95001_MOESM1_ESM.pdf]

Water removal during automated peritoneal dialysis assessed by remote patient monitoring and modelling of peritoneal tissue hydration

Joanna Stachowska-Pietka, Beata Naumnik, Ewa Suchowierska, Rafael Gomez, Jacek Waniewski, Bengt Lindholm

SUPPLEMENTARY MATERIAL

Peritoneal transport model

The spatially distributed model of peritoneal transport [1,2] was used to simulate changes - as a function of distance from the peritoneal cavity - of peritoneal tissue characteristics such as interstitial pressure, tissue hydration (formally interstitial fluid void volume ratio), and solute interstitial concentration, based in part on reported changes of intraperitoneal volume and solute concentrations occurring during a peritoneal dialysis dwell [3,4]. We explored intraperitoneal volume and pressure equilibration and associated changes of pressure and hydration of the peritoneal tissue close to its surface in contact with dialysate during the whole peritoneal dialysis exchange. The accumulation of water in the peritoneal tissue was calculated as the aggregated (over distance from the peritoneal cavity) difference between volume profiles of tissue hydration, i.e., for the first APD cycle (C1) as the difference between the end of the first dwell of APD exchange vs. end of day exchange, and for the second APD cycle (C2) as the difference between volume profiles obtained at the end of second APD dwell minus the volume at the end of C1. To obtain total water accumulation during the investigated periods, the values (estimated by the model per unit surface area) were multiplied by the corresponding effective peritoneal surface area that remained in contact with dialysate.

The peritoneal transport system was modelled with blood and lymph capillaries spatially distributed within the peritoneal tissue space. The three-pore model with ultra-small (aquaporins), small and large pores was used to describe transport across the blood capillary wall [5,6]; a similar model was proposed to describe transport across the whole peritoneal barrier by Rippe et al [7]. In this approach, water and solute peritoneal transport through the tissue depends not only on the local pressures, tissue hydration and concentrations that drive each transport component but also on the local properties of the tissue that are changing due to physiological responses to the ongoing treatment. The variability of the effective peritoneal surface area (EPSA, i.e., the surface that remains in contact with the dialysate) caused by the intraperitoneal volume changes was taken into account using a function

previously described by Keshaviah et al [8,9]. The peritoneal tissue adapts to the increased intraperitoneal pressure and volume caused by the start of APD session by increased hydration of the peritoneal tissue that remains in contact with dialysate within a thin layer, close to the peritoneal cavity. However, in case of the APD_{DD} regime, the previously overhydrated peritoneal tissue loses contact with dialysate during the day exchange. This results in local leakage of fluid into the peritoneal cavity from parts of the peritoneal tissue that remain without contact with dialysate leading towards a new physiological equilibrium with less hydration. The above-mentioned process occurring during the daytime exchange was taken into account, assuming free outflow of fluid from the parts of the tissue that were no longer in contact with dialysate.

To avoid overestimation of the peritoneal absorption (especially during the infusion and drainage procedures), a more detailed description was applied. Namely, two types of the overall peritoneal fluid absorption was considered: direct absorption by diaphragmatic lymphatics that are open to the peritoneal cavity (assumed to account for 30% of total peritoneal absorption and not present if the intraperitoneal volume is lower than 500 mL) and absorption to the peritoneal tissue, that accounts for 70% of total peritoneal absorption and is proportional to the EPSA [10]. The transport of solutes (glucose, sodium, urea, and creatinine) was also taken into account, as previously proposed [1]; data not presented here.

Computer simulations were performed for a typical patient undergoing standard APD_{DD} regime with 6 APD cycles for 90 min each with infused volume of 2 L of glucose 1.36%, followed by dry day albeit with infusion of 100 mL of glucose 1.36% to avoid pain associated with completely empty abdominal cavity. The peritoneal membrane characteristics was taken for a typical patient undergoing CAPD with 2.27% glucose solution based on results of fitting parameters for clinical data published by Heimbürger et al. [11], cited in [2]. The residual volume was assumed to be 300 mL and the maximal rate of peritoneal absorption equal to 1 mL/min. Simulations of three days of dialysis exchanges were performed to assure complete adaptation of the peritoneal tissue to the treatment. Results are presented in **Figures 2** and **3**.

Additional numerical simulations

The discrepancy observed in APD_{DD} between first and remaining APD cycles would conceivably become less apparent if a larger volume of dialysis fluid was infused during the daytime because then a larger fraction of the peritoneal tissue would remain in contact also during the daytime exchange resulting in less decline of peritoneal tissue hydration. Nevertheless, numerical simulations performed for the same, typical patient using instead of APD_{DD} a wet day APD_{WD} regime with 2 L of infused volume of glucose 1.36%, revealed that inter-cycle changes of tissue hydration, although less pronounced, might

be still present in APD_{WD}, see **Figures S1**. However, the obtained results cannot be directly compared with the actual clinical data of the studied APD_{WD} group, which differed from the APD_{DD} group and all except one of the APD_{WD} patients received icodextrin-based dialysis fluid for the daytime exchange. One may speculate, that for patients using icodextrin for the daytime exchange, higher UF associated with usage of icodextrin [12-16] would result in the even smaller decrease of tissue hydration and therefore lower discrepancy in water removal between the first vs. consecutive APD cycles than obtained in our simulations with 1.36% glucose for the wet day.

Since water removal depends on the glucose tonicity, higher glucose concentration used for the daytime exchange would result in higher ultrafiltration, larger peritoneal surface area remaining in contact with dialysis fluid during the daytime exchange, lower leakage of water from the tissue that loses its contact with peritoneal fluid, and, in consequence, less pronounced decrease of peritoneal tissue hydration towards a physiological state. However, due to the low infused volume used for the daytime exchange in APD_{DD} the impact of fluid tonicity would remain within measurements error. Numerical simulations showed that an increase of glucose concentration in dialysate used for the daytime exchange from 1.36% to 2.27%, provides only marginally higher daytime UF (-98 mL vs. -110 mL for daytime use of 1.36% glucose) and water removal for the whole APD session (485 mL for glucose 2.27% vs. 458 mL for daytime use of 1.36% glucose) related to changes in net UF during the first cycle of APD session), see **Figure S2 left panel**. The low impact of this change (from 1.36% to 2.27% during daytime exchange) on daily water removal results in negligible decrease of the peritoneal surface area that remains without contact with dialysate and therefore negligible change in peritoneal hydration state day as compared to use of 1.36% during the daytime exchange, **Figure S2 right panel**.

Supplemental Figures

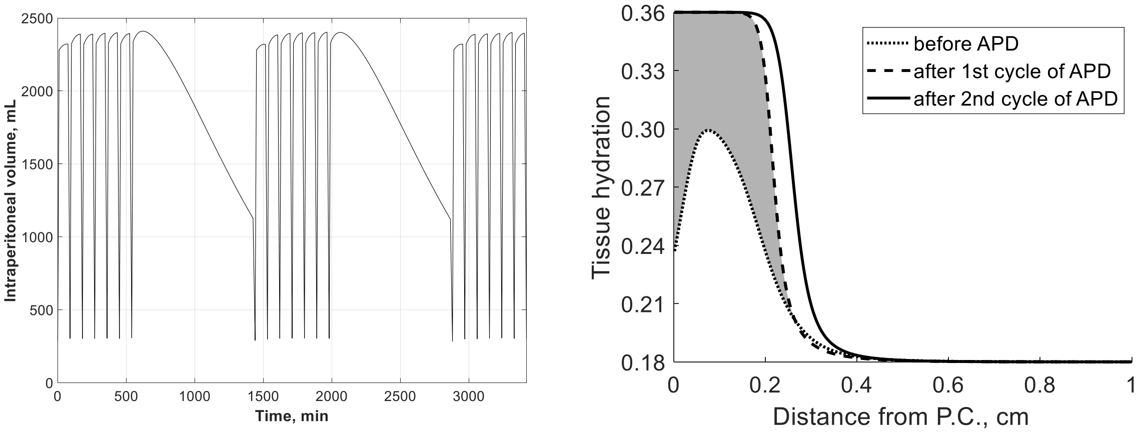

Figure S1. Numerical simulations performed for a typical patient using the APD_{WD} regime. **Left panel:** Intraperitoneal volume profiles as a function of time during three consecutive days. **Right panel:** Hydration of peritoneal tissue in contact with dialysis fluid during APD session predicted by the model as a function of distance from the peritoneal cavity (P.C.): dotted line - before starting of APD session after a wet day, dashed line – after the first cycle of APD session, solid line – after the second cycle of APD session. All simulations were performed for three APD sessions (consisting of six 90-minute exchanges with 2 L of glucose 1.36%) interrupted by wet day regime with infused volume of 2 L of glucose 1.36%.

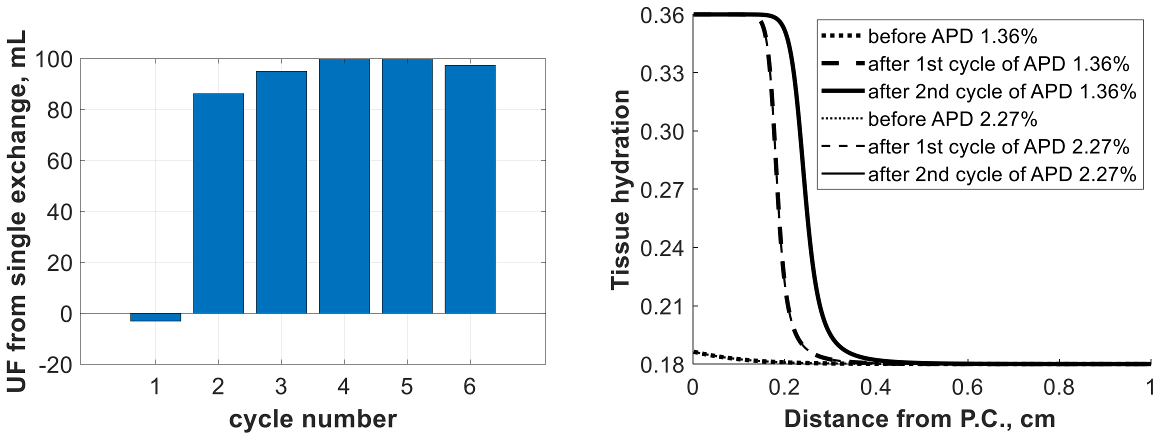

Figure S2. Numerical simulations performed for a typical patient using the APD_{DD} regime with daytime exchange with 1.36% or 2.27% glucose. **Left panel:** The net ultrafiltration per every single exchange of APD session preceded by daytime exchange with 2.27% glucose. **Right panel:** Peritoneal tissue hydration during APD session with dry day regime with glucose 1.36% (solid line) and 2.27% (thin line) used for a daytime exchange: dotted line - after a wet day and before APD session, dashed line – after the first cycle of APD, solid line – after the second cycle of APD session. Infusion of 100 mL of glucose 1.36% or 2.27% was assumed in the case of a daytime exchange and the APD session was simulated with 6 cycles of 90 minutes each with an infusion of 2 L of glucose 1.36%.

References

1 Stachowska-Pietka, J., Poleszczuk, J., Flessner, M. F., Lindholm, B. & Waniewski, J. Alterations of peritoneal transport characteristics in dialysis patients with ultrafiltration failure: tissue and capillary components. *Nephrol Dial Transplant* **34**, 864-870, doi:10.1093/ndt/gfy313 (2019).

2 Stachowska-Pietka, J., Waniewski, J., Flessner, M. F. & Lindholm, B. Computer simulations of osmotic ultrafiltration and small-solute transport in peritoneal dialysis: a spatially distributed approach. *Am J Physiol Renal Physiol* **302**, F1331-1341, doi:10.1152/ajprenal.00301.2011 (2012).

3 Fischbach, M., Zaloszyc, A., Schaefer, B. & Schmitt, C. Adapted automated peritoneal dialysis. *Adv Perit Dial* **30**, 94-97 (2014).

4 Fischbach, M., Zaloszyc, A., Schaefer, B. & Schmitt, C. P. Optimizing peritoneal dialysis prescription for volume control: the importance of varying dwell time and dwell volume. *Pediatr Nephrol* **29**, 1321-1327, doi:10.1007/s00467-013-2573-x (2014).

5 Watson, P. D. Permeability of cat skeletal muscle capillaries to small solutes. *Am J Physiol* **268**, H184-193, doi:10.1152/ajpheart.1995.268.1.H184 (1995).

6 Wolf, M. B. Determination of the magnitude of the water-exclusive pathway in cat skeletal muscle microvasculature. *Microcirculation* **3**, 59-73, doi:10.3109/10739689609146783 (1996).
7 Rippe, B., Venturoli, D., Simonsen, O. & de Arteaga, J. Fluid and electrolyte transport across the peritoneal membrane during CAPD according to the three-pore model. *Perit Dial Int* **24**, 10-27 (2004).
8 Keshaviah, P., Emerson, P. F., Vonesh, E. F. & Brandes, J. C. Relationship between body size, fill volume, and mass transfer area coefficient in peritoneal dialysis. *J Am Soc Nephrol* **4**, 1820-1826 (1994).
9 Oberg, C. M. & Rippe, B. Optimizing Automated Peritoneal Dialysis Using an Extended 3-Pore Model. *Kidney Int Rep* **2**, 943-951, doi:10.1016/j.ekir.2017.04.010 (2017).
10 Heimburger, O., Waniewski, J., Werynski, A., Park, M. S. & Lindholm, B. Lyphatic absorption in CAPD patients with loss of ultrafiltration capacity. *Blood Purif* **13**, 327-339, doi:10.1159/000170217 (1995).
11 Heimburger, O., Waniewski, J., Werynski, A. & Lindholm, B. A quantitative description of solute and fluid transport during peritoneal dialysis. *Kidney Int* **41**, 1320-1332, doi:10.1038/ki.1992.196 (1992).
12 Olszowska, A. *et al.* Long Peritoneal Dialysis Dwells With Icodextrin: Kinetics of Transperitoneal Fluid and Polyglucose Transport. *Front Physiol* **10**, 1326, doi:10.3389/fphys.2019.01326 (2019).
13 Olszowska, A. *et al.* The kinetics of water transperitoneal transport during long-term peritoneal dialysis performed using icodextrin dialysis fluid. *Pol Arch Med Wewn* **119**, 305-310 (2009).
14 Leypoldt, J. K., Hoff, C. M., Akonur, A. & Holmes, C. J. Low-Polydispersity Glucose Polymers as Osmotic Agents for Peritoneal Dialysis. *Perit Dial Int* **35**, 428-435, doi:10.3747/pdi.2013.00232 (2015).
15 Goossen, K. *et al.* Icodextrin Versus Glucose Solutions for the Once-Daily Long Dwell in Peritoneal Dialysis: An Enriched Systematic Review and Meta-analysis of Randomized Controlled Trials. *Am J Kidney Dis* **75**, 830-846, doi:10.1053/j.ajkd.2019.10.004 (2020).
16 Mistry, C. D., Gokal, R. & Peers, E. A randomized multicenter clinical trial comparing isosmolar icodextrin with hyperosmolar glucose solutions in CAPD. MIDAS Study Group. Multicenter Investigation of Icodextrin in Ambulatory Peritoneal Dialysis. *Kidney Int* **46**, 496-503, doi:10.1038/ki.1994.300 (1994).
